# Supplementary material for: Mimicking the Intestinal Host–Pathogen Interactions in a 3D In Vitro Model: The Role of the Mucus Layer
Source: Pharmaceutics. 2022 Jul 26;14(8):1552. doi: 10.3390/pharmaceutics14081552 (PMC9331835; doi:10.3390/pharmaceutics14081552)
Supplement: Supplementary file 1 [file pharmaceutics-14-01552-s001.zip › pharmaceutics-1809272-supplementary.pdf]

Mimicking the Intestinal Host–Pathogen Interactions in a 3D in Vitro Model: The Role of the Mucus Layer

María García-Díaz [1,*], Maria del Mar Cendra [1,†], Raquel Alonso-Roman [1,‡], María Urdániz [1], Eduard Torrents [1,2] and Elena Martínez [1,3,4,*]

[1] Institute for Bioengineering of Catalonia (IBEC), The Barcelona Institute of Science and Technology (BIST), 08028 Barcelona, Spain; mar.cendra@upc.edu (M.d.M.C.); raquel.roman@leibniz-hki.de (R.A.-R.); murdaniz@ibecbarcelona.eu (M.U.); etorrents@ibecbarcelona.eu (E.T.)

[2] Microbiology Section, Department of Genetics, Microbiology, and Statistics, Biology Faculty, University of Barcelona, 08028 Barcelona, Spain

[3] Centro de Investigación Biomédica en Red de Bioingeniería, Biomateriales y Nanomedicina (CIBER-BBN), 28029 Madrid, Spain

[4] Department of Electronics and Biomedical Engineering, University of Barcelona, 08028 Barcelona, Spain

* Correspondence: mgarcia@ibecbarcelona.eu (M.G.-D.); emartinez@ibecbarcelona.eu (E.M.)

† Current address: Department of Agri-Food Engineering and Biotechnology, Polytechnic University of Catalonia, 08860 Castelldefels, Spain.

‡ Current address: Department of Microbial Pathogenicity Mechanisms, Leibniz Institute for Natural Product Research and Infection Biology, Hans Knoell Institute, 07745 Jena, German

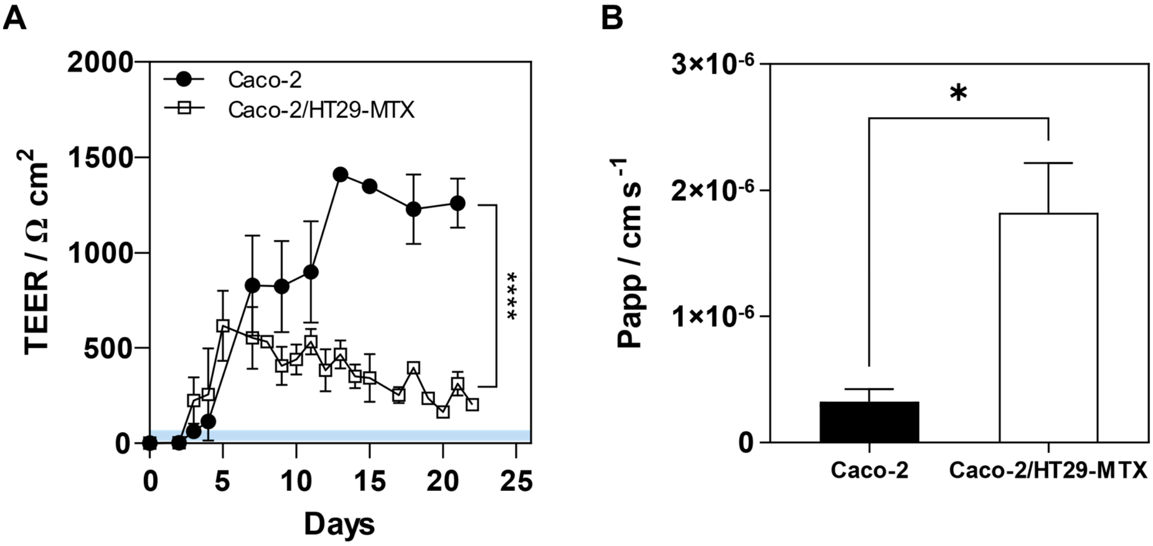

Figure S1. (**A**) Transepithelial electrical resistance (TEER) of the Caco-2 monoculture (black dots) or the Caco-2/HT29-MTX co-culture (open squares) on top of 2D Transwell membranes. The blue shadow indicates the range of the reported TEER values of the human small intestine (Le Ferrec et al., 2001). (**B**) Apparent permeability (Papp) values of the FD4 model compound across the 2D models. Mean ± SEM, $n > 8$, * ($p < 0.05$), **** ($p < 0.0001$).

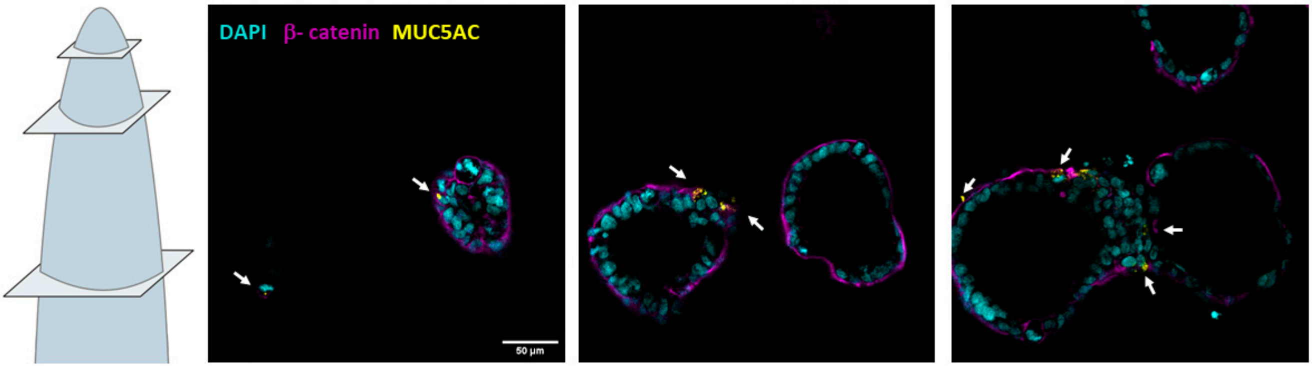

Figure S2. Detailed cross-sections of representative microsctructures covered with Caco-2/HT29-MTX co-culture grown on top of the villus-like microstructures. Views from the tip, half-height and bottom of the scaffold. Nuclei are shown in cyan, β-catenin in magenta and MUC5AC in yellow. White arrows indicate the distribution of the goblet-like cells. Scale bar = 50 µm

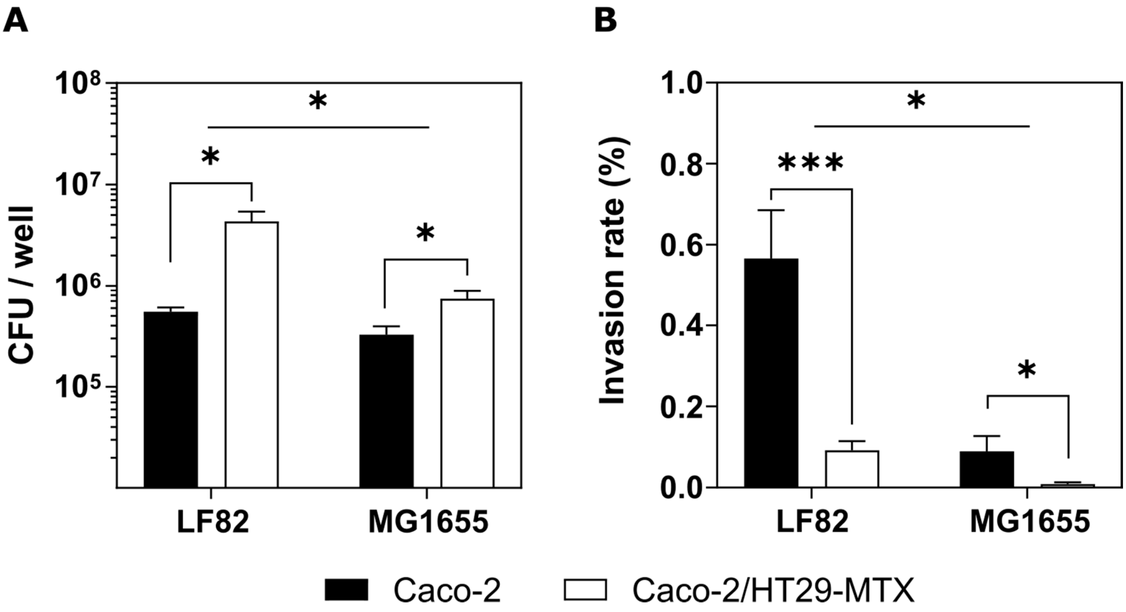

Figure S3. Quantification of adherent (**A**) or invaded (**B**) bacteria (*E. coli* LF82 or MG1655 strains) to the 2D Caco-2 monoculture (black bars) or the 3D Caco-2/HT29-MTX co-culture (white bars) after 3 h of infection. The bacterial adhesion is expressed in CFU/well. The bacterial invasion is determined after 1 h gentamicin incubation and expressed as the percentage of the adhered bacteria. Mean ± SEM of at least two independent experiments with 3 replicates, * ($p < 0.05$), *** ($p < 0.001$).

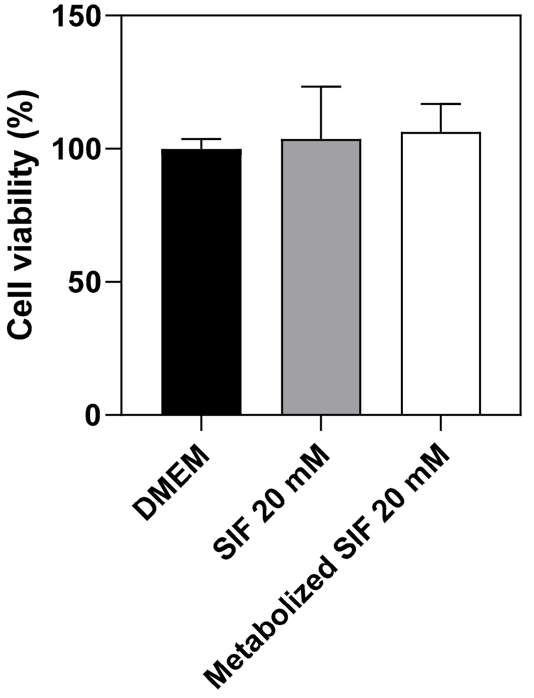

Figure S4. Cellular viability of the 3D Caco-2/HT29-MTX co-culture model after 3 h incubation with SIF containing 20 mM sodium taurocholate and 5 mM L-α-phosphatidylcholine. To address the toxicity of secondary bile salts, produced by bacterial metabolism, SIF was incubated in the presence of LF82 during 3h. The metabolized medium was centrifuged and filtered through 0.22 µm pore size before adding to the 3D co-culture model. Mean ± SEM of two biological replicates.

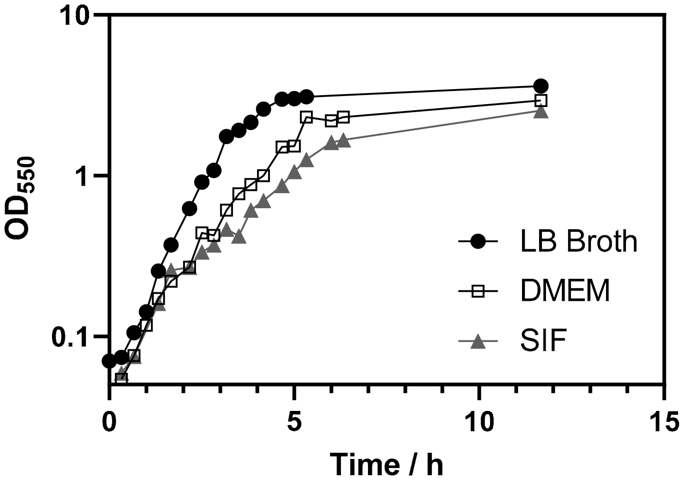

Figure S5. Bacterial growth kinetics of *E. coli* LF82 in different media.
